# Multiplex Strand Invasion Based Amplification (mSIBA) assay for detection of *Chlamydia trachomatis* and *Neisseria gonorrhoeae*

**DOI:** 10.1038/srep20487

**Published:** 2016-02-03

**Authors:** Kevin E. Eboigbodin, Mark J. Hoser

**Affiliations:** 1 Research and Development, Orion Diagnostica, Espoo, Finland; 2 Molecular Biology, GeneForm Technologies, Broadstairs, United Kingdom

## Abstract

Nucleic acid amplification tests have become a common method for diagnosis of STIs due to their improved sensitivity over immunoassays and traditional culture-based methods. Isothermal nucleic acid amplification methods offer significant advantages over polymerase chain reaction (PCR) because they do not require sophisticated instruments needed for thermal cycling of PCR. We recently reported a novel isothermal nucleic acid amplification method, Strand Invasion-Based Amplification (SIBA), which exhibited high analytical sensitivity and specificity for amplification of DNA. However, because the reactions were detected using an intercalating dye, this method was only suitable for amplifying a single genomic target. Here, we report the development of multiplexed SIBA (mSIBA) that allows simultaneous detection of *Chlamydia trachomatis* (CT), *Neisseria gonorrhoeae* (NG), and an internal control in the same reaction tube. SIBA is compatible with probes, allowing the detection of multiple DNA targets in the same reaction tube. The IC was developed to assess the quality of the isolated DNA and the integrity of the enzyme system, as well as to test oligonucleotides. The mSIBA assay retained high analytical sensitivity and specificity for the detection of CT and NG. The development of mSIBA enables rapid screening for CT and NG within point-of-care or central laboratory settings.

*Chlamydia trachomatis* (CT) and *Neisseria gonorrhoeae* (NG) infections are among the most common causative agents of sexually transmitted infections (STIs)^1^,^2^. Nucleic acid amplification tests (NAATs) are now the method of choice in clinical laboratories worldwide for routine diagnosis of CT and NG. NAATs offer superior sensitivity and specificity in comparison with immunoassays and traditional culture-based methods^3^,^4^. The latter are often very time-consuming, and also rely on the presence of viable organisms in the specimen. Because of the high analytical sensitivity of NAATs, direct detection of CT or NG can be performed using non-invasive specimens such as urine^5^.

In an attempt to reduce the sample processing time and overall cost of NAATs, it is often desirable to perform multiplexed tests to simultaneously detect two or more genomic targets or organisms in a single reaction tube. Several commercially available multiplexed NAATs can simultaneously detect of CT and NG, which are frequently comorbid. These tests also include an internal control (IC) for assessing potential sample-related inhibition. The existing tests are often based on multiplexed polymerase chain reaction (PCR), using specific primers and dual-labeled probes for CT, NG, and IC in a single reaction tube. Despite the emergence of isothermal nucleic acid amplification platforms that obviate the use of sophisticated thermal cyclers, PCR still remains the most common platform used for NAAT methods.

We previously described a novel isothermal nucleic acid amplification method, Strand Invasion-Based Amplification (SIBA), with high analytical sensitivity and specificity^6^. SIBA produces only target-specific reaction products, which can be detected using intercalating dyes alone. However, such dyes only detect total double-stranded DNA, limiting their use to detection of single targets. Here, we describe the development of an IC and a probe-based method that allows SIBA reactions to be multiplexed. The IC assay was developed to allow more precise assessment of sample-derived reaction inhibition. We also demonstrate the use of this multiplexed method in the simultaneous detection of CT, NG, and IC in a single reaction tube. We compared the performance of SIBA to those of two existing DNA amplification methods, real-time PCR and loop-mediated isothermal amplification (LAMP).

## Results

### Sensitivity and specificity of SIBA, LAMP and PCR singleplex assays

We developed singleplexed SIBA assays that detected a specific sequence from the CT cryptic plasmid or NG-*porA,* and compared these assays with previously published LAMP and PCR assays to detect the same targets. We also developed an in-house LAMP assay to detect specific sequences from *porA*, because the published LAMP assay was designed to detect NG *ORF1* of the glutamine synthetase *(glnA)* gene^7^. Both SIBA and LAMP assays were detected using intercalating dyes, because such methods do not rely on target-specific probes for detection of the target amplicon. The CT and NG PCR assays were detected using the Taqman probe chemistry^8^. The sensitivities of the SIBA, LAMP, and PCR assays for CT and NG were evaluated in at least three independent experiments by serially diluting the positive control DNA (constructed CT-plasmid CTPlas-pUC57 for CT assay and ZeptoMetrix NG control for NG assay) from 2 × 10^5^ copies to 2 copies in quadruplicate (Tables 1 and 2). All three DNA amplification methods (SIBA, LAMP, and PCR) were sensitive at the level of 20 copies per reaction. All three methods occasionally detected as few as 2 copies of target DNA, probably due to inconsistencies in the actual amount of DNA present at such low dilutions.

The specificities of all three amplification methods for the detection of CT or NG were challenged by the addition of DNA pooled from 10 different unrelated microorganisms (10^3^ DNA copies per reaction). CT and NG assays were further challenged with 10^5^ copies of DNA from non-gonococcal *Neisseria* strains (*N. meningitidis*, *N. flavescens*, *N. subflava*, and *N. sicca*) with genomes closely related to that of NG. Neither SIBA, LAMP, nor PCR assay detected DNA pooled from 10 different unrelated microorganisms in either the CT or NG assay, and neither SIBA nor PCR NG assay detected DNA from non-gonococcal *Neisseria* strains. However, both the in-house and published LAMP assays detected some of the non-gonococcal *Neisseria* strains. This finding suggests that both SIBA and PCR offer some advantage in assays in which exclusive detection of closely related targets is desired. (Supporting information S1; alignment map of SIBA, LAMP and PCR NG primers in relation to porA gene from *Neisseria gonorrhoeae* and *Neisseria meningitidis*).

### Incorporation of target-specific probes in SIBA

Because SIBA typically produces specific reaction products, intercalating dyes such as SYBR Green 1 are sufficient to detect amplification in SIBA reactions^6^. However, such dyes only detect total dsDNA, limiting their use to detection of single targets. To address this shortcoming, we developed a probe-based method that could be used for mSIBA reactions. Short single-stranded dual-labeled probes (12–14 nucleotides) containing a mixture of DNA and LNA bases were designed to be complementary to the downstream region of the target DNA and homologous to the 5′-end of the reverse primer. The probes were labeled with a fluorophore at the 5′-end and a quencher at the 3′-end, such that the probe’s fluorescence signal increases upon hybridisation to its complementary sequence. The inclusion of a short sequence of LNA bases in the middle of the probe increased the melting temperature so that it could stably bind the complementary sequence under SIBA reaction conditions^9^. The inclusion of LNA bases also abolished the effect of including recombinase and single-stranded DNA binding protein (T4 gp32) in the SIBA reaction, which would otherwise simulate a double-stranded configuration of the probes and elicit a false signal (Supporting information S2)^10^.

**Figure 1:**
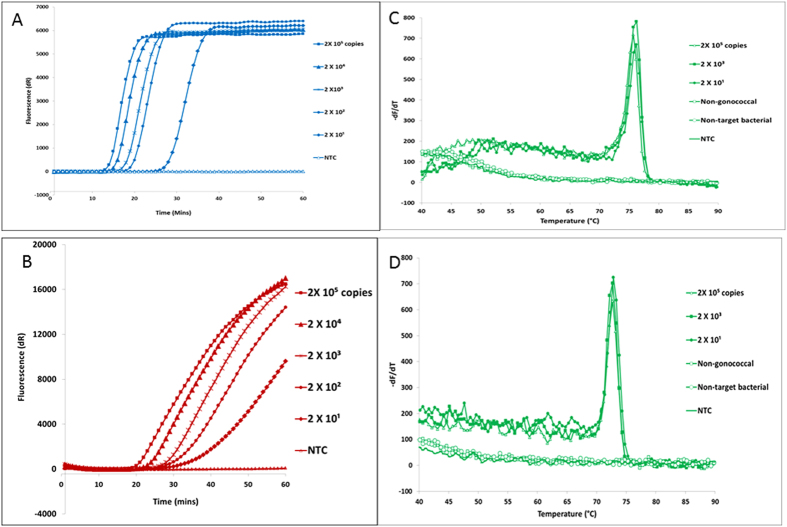
Detection of SIBA *Neisseria gonorrhoeae* (NG) and *Chlamydia trachomatis* (CT) using LNA probes. (**A**) *Neisseria gonorrhoeae.* (**B**) *Chlamydia trachomatis*. Probes for NG and CT assays were labeled with Cy5 and ROX, respectively. (**C**) Corresponding melting curve analysis for NG assay (**D**) corresponding melting curve analysis for CT assay. 2 × 10^4^ and 2 × 10^2^ NG and CT reactions were omitted from Fig. 1C,D for the sake of clarity.

**Figure 2:**
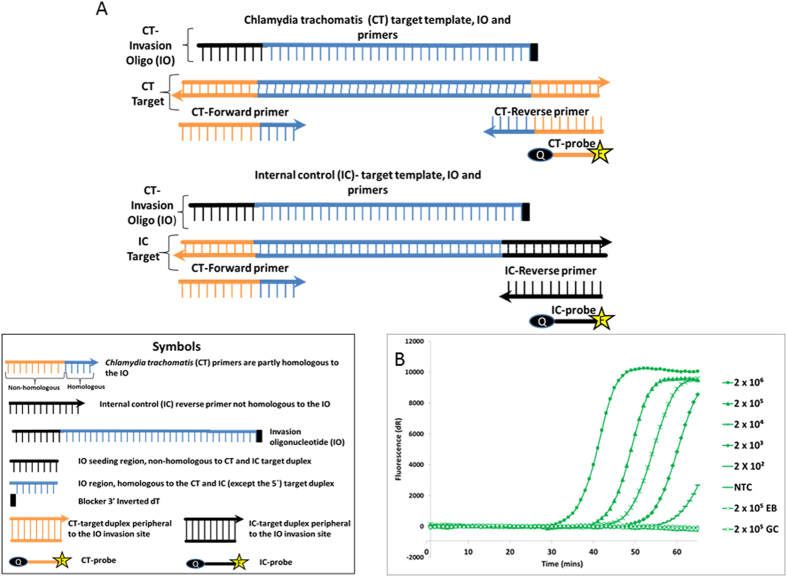
Internal control (IC) development. IC control assay uses the same forward primer and IO as the *Chlamydia trachomatis* (CT) assay. (**A**) Configuration of oligonucleotides and probes used in the IC and CT assays. (**B**) Amplification of the IC control assay using 2 × 10^6^ to 2 × 10^2^ copies of IC template. IC reactions were detected with a FAM-labeled LNA probe.

We designed and tested probes for the SIBA CT and NG assays, which were previously detected using the intercalating dye SYBR Green 1. The CT probe was labeled with ROX and Iowa black FQ quencher, whereas the NG probe was labeled with Cy5 and Iowa black RQ quencher. The amplification signal profile of these assays when detected with either SYBR Green 1 or the labeled probes can be seen in Fig. 1B. The increase in fluorophore signal, attributed to binding of the probes to the amplicon, can be seen only in reactions containing the target DNA. The probes produced robust signals without compromising the efficiency of amplification (Fig. 1). Although a high concentration of probes might compete with the downstream primer, the probe concentration used in this study was not inhibitory, and the SIBA reactions were still efficient in the presence of the dual-labeled probes. This is partially due to the ability of the primer 3′- end to bind the target DNA even in the presence of a probe (Fig. 2A).

### IC development

Next, we developed an IC assay to assess possible sample-derived inhibition. This was achieved by constructing an artificial target template with a sequence assay identical to the CT target, but differing in its downstream region. Therefore, the IC assay shared the same forward primer and IO as the CT assay, and differed from the CT assay only in the reverse primer and probe used (Fig. 2A). The sensitivity of the IC assay was established by serially diluting the IC DNA template from 2 × 10^6^ to 200 copies per reaction. The IC assay was performed under SIBA reaction conditions using the CT-forward primer, CT-IO, IC-reverse primer, and IC-probe (FAM), and real-time amplification was measured by monitoring the fluorescence produced by the probe. The increase in fluorescent signal was only detected in reactions containing IC template (Fig. 2B), and the IC assay did not cross-react with either the CT or the NG template. Likewise, the CT or the NG assays are unable to amplify the IC template.

### Performance of multiplexed SIBA CT and NG assay

**Figure 3:**
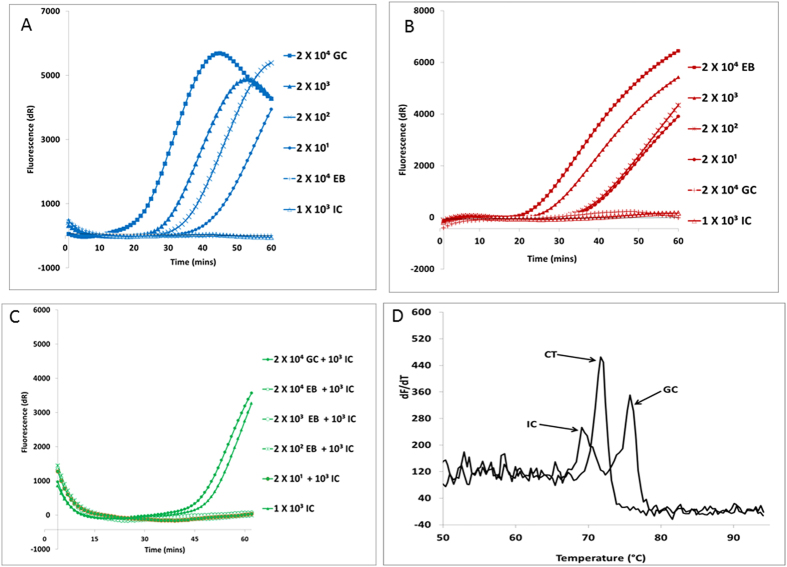
Multiplexed *Chlamydia trachomatis* (CT), *Neisseria gonorrhoeae* (NG), and internal control (IC) assay. (**A**) Detection of NG using Cy5-labeled LNA probe. (**A**) Detection of CT using ROX-labeled LNA probe. (**C**) IC control using FAM-labeled LNA probe. (**D**) Melt curve of CT, NG, and IC reaction product detection with SYBR Green.

We then developed a multiplexed SIBA assay for the detection of CT and NG in a single reaction tube. The performance of the multiplexed SIBA assay was compared with previously validated multiplexed PCR assays for the detection of CT and NG^8^,^11^. The SIBA multiplexed reaction, which consisted of a 3-plex reaction of the CT, NG, and IC assays described in Figs. 1 and 2, was performed using lysates from CT elementary bodies (EB) and NG cells. The sensitivity of the multiplexed assay was evaluated by serially diluting the lysate containing from 2 × 10^4^ to 2 EB or CFU of NG cells per reaction. Each reaction also contained 1000 copies of the IC template. The multiplexed CT/NG/IC assay reproducibly amplified and detected as few as 20 EB or 20 NG cells per reaction (Fig. 3). Although the assay was also capable of amplifying and detecting 2 EB or 2 NG cells per reaction, the variability at these dilutions was high, again probably due to inconsistencies in the actual amount of DNA present.

The IC template was detected in reactions that did not contain CT EB. The CT assay was designed to amplify more efficiently than the IC assay. The presence of the EB in the reaction and consequent positive CT test result leads to the consumption of CT-forward primers, which are required for IC template amplification. Therefore, the IC is a conditional control that ensures the negative reaction is not a result of sample inhibition; consequently, it is not needed in a positive assay. The specificity of the multiplexed CT/NG/IC assay was established by challenging the reaction with a pool of cell lysate from 10 different unrelated microorganisms (see Table 3 for a list of the microorganisms used). Another pool of cell lysate from four different non-gonococcal *Neisseria* strains was also used to establish assay specificity. Neither the unrelated microorganisms nor the non-gonococcal *Neisseria* strains cross-reacted with the multiplexed CT/NG/IC assay. Furthermore, the EB and NG could only be detected via their corresponding probe channels (ROX and Cy5, respectively). The multiplexed CT/NG/IC assay was also used to detect EB and NG in presence of nucleic acids from 14 unrelated microorganisms (10^3^ copies from each microorganism per reaction, Table 3). No reduction in assay efficiency was observed. The assay was also able to detect CT and NG cells from a low positive clinical control specimen (The amplification curves can be seen in figure supporting information S3). These results demonstrate that the multiplexed CT/NG/IC assay was highly specific. The analytical sensitivity and specificity of the multiplexed CT/NG/IC were found to be similar to the previously validated multiplexed PCR assay for the detection of CT and NG^8^,^11^. However, the mSIBA assay displayed a shorter detection time than the multiplexed PCR assay.

**Figure 4:**
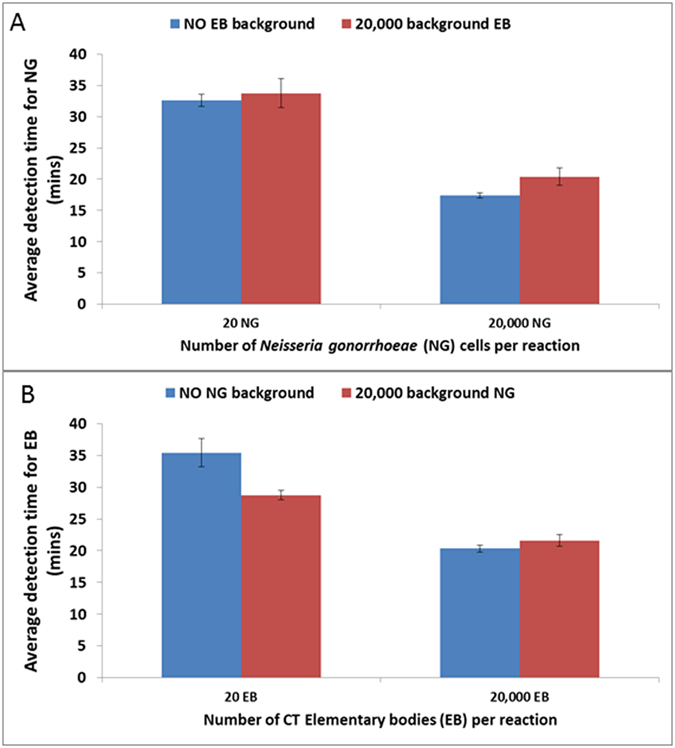
Competition between *Chlamydia trachomatis* (CT) and *Neisseria gonorrhoeae* (NG) in multiplexed reactions. (**A**) Detection of CT elementary body (EB) in the presence of a high background of NG. (**B**) Detection of NG in presence of a high background of CT. Average detection from eight replicates. The detection time reported was the time at which the probe fluorescent signal exceeded the background signal.

We further evaluated the multiplexed assay for possible competition between CT and NG detection. In particular, we assessed the ability of the assay to detect low levels of EB in the presence of high levels of NG cells, and vice versa (Fig. 4). The presence of high levels of NG cells in the reaction did not result in any significant reduction in amplification from a small number of EBs. Similarly, the presence of large numbers of EBs in the reaction did not significantly reduce amplification from small numbers of NG cells. This suggests that the SIBA multiplexed CT/NG assay would be suitable for detecting these targets in patient samples co-infected with CT and NG.

## Discussion

The results of this study demonstrated the ability of SIBA to detect NG and CT together in the presence of an IC in a single reaction tube. SIBA was previously shown to exhibit high analytical sensitivity and specificity for amplification of genomic DNA^6^. However, because the reactions were detected using an intercalating dye, the method was only suitable for amplifying a single genomic target. SIBA was also found to be compatible with probes and ICs such that multiple target DNAs could be simultaneously amplified and detected in a single reaction tube without loss of assay integrity. Furthermore, the multiplexed assays performed with a sensitivity and specificity similar to those of the singleplexed assay.

SIBA relies on a recombinase (UvsX) and a single-stranded binding protein for dissociation of the target duplex. This allows target-specific primers to bind and extend the target via the action of a DNA polymerase. The use of dual-labeled probes in SIBA also presented some challenges in assay design because of the presence of single-strand binding (SSB) proteins in the reaction mixture. SSB proteins bind to ssDNA, simulating a double-stranded configuration in terms of the quencher/fluorophore-labeled probes, limiting their ability to discriminate between a complementary and non-complementary target template. Because SSB proteins do not bind efficiently to LNA, this problem was solved by incorporating LNA bases in the dual-labeled probes^10^.

The ICs included in most nucleic acid amplification methods are often housekeeping genes, but these do not test for the functionality of the primers used in the test reaction. By contrast, the ICs developed here can assess DNA isolation and the integrity of the enzyme system, as well as test oligonucleotides. Previous studies showed that the efficiency of the SIBA reaction is influenced by the length of the target region peripheral to IO invasion, which forms a part of the primer binding site^6^. A longer region, typically greater than 14 bp, leads to a reduction in amplification efficiency. Based on these previous findings, we designed the IC assay to have a peripheral region longer than 14 bp to ensure that the assay was less efficient than the CT or NG assays. This approach ensures that the IC assay does not compete with the CT or NG assays when the reactions are performed in the same reaction tube. Consequently, the detection time of the IC assay was longer than those of the CT and NG assays (Fig. 2B). Because a low level of IC template was used in the multiplex assay, the IC template was only detected after small numbers of copies of CT or NG were detected in the reaction. This further ensured that the IC assay did not compete with the CT assay for primers and IO. Although the IC assay was designed to use the same forward primer and IO as the CT assay, it can also be constructed to use the same forward primer and IO as the NG assay. This makes it possible to have multiple ICs (one for each test analyte) in the same reaction tube. Such an approach could further improve assay robustness and reliability.

The multiplexed SIBA CT/NG/IC assay could only be compared directly with a previously published multiplexed PCR assay. To our knowledge, no multiplexed LAMP assay for the detection of CT and NG has been reported in the literature. CT and NG detection have, however, been multiplexed by a number of other nucleic acid amplification methods^5^,^12^,^13^. The unavailability of a multiplex CT/NG LAMP assay may be due to the use of intercalating dye-based detection^14^ or turbidimetry^15^ in LAMP assays; these detection methods are limited to monitoring of a single target. Furthermore, multiplexing in LAMP could also prove problematic because of the use of numerous large primers that lead to unwanted primer interactions, resulting in generation of unwanted false positives. In the case of SIBA, the specificity of the reaction is mainly driven by the non-extendible IO rather than the primers. A template that differs by two or more nucleotides in the region homologous to the IO will amplify poorly or not at all^6^, allowing multiple primers to be included in the same reaction for the purpose of multiplexing. This may also be the reason why SIBA NG assay did not cross react with other commensal *Neisseria* strains, a problem seen in some NAAT commercial kits^16^. Although real-time PCR is a robust NAAT platform for multiplexing several diagnostic analytes, the requirement for thermal cyclers limits the use of PCR for field or point-of-care applications. In this study, we used a qPCR device for convenience to perform multiple reaction conditions and replicates. Several small and portable low-cost devices have been developed for the fluorescent detection of common isothermal DNA amplification reactions^17^. These devices can also be used for the detection of mSIBA reactions and are more convenient for field or point-of-care applications.

CT and NG are major pathogens, and over 6 million tests were performed in 2007 in the USA^18^. Effective and early diagnosis plays an important role in treatment and preventing spread of these infections. It is desirable to perform routine screening in physicians’ offices or polyclinics, rather than in central laboratory settings. Therefore, the development of a robust multiplex isothermal amplification method could enable detection of CT and NG within these settings, because such methods would be compatible with low-cost and low-footprint devices.

## Materials and Methods

### Materials and reagents

The oligonucleotides used in this study were purchased from Eurofins Genomics (Germany) or Integrated DNA Technologies (Belgium) and purified by the manufacturer using reverse-phase HPLC (for primers and probes) and PAGE (for the invasion oligonucleotides). Sequences are shown in Table 4. Sucrose and sucrose phosphorylase were purchased from Sigma-Aldrich (USA).

### Bacterial strains and DNA preparation

A 993-bp template harboring positions 1020–1380, 3315–3785, and 6530–6690 from CT plasmid pCHL1 (NC_001372) was cloned into plasmid pUC57 (Eurofins, Germany). The plasmid was quantified by UV absorbance at 260 nm using a NanoDrop 1000 Spectrophotometer (Thermo Scientific, Wilmington, DE) and the number of copies was calculated. Number of copies = (amount X 6.022×10^23^)/(length X 1x10^9^ × 650). The constructed plasmid (CTPlas-pUC57) served as the positive control template for establishing the analytical sensitivity of SIBA, LAMP, and PCR singleplexed CT assays. Commercially quantitated NG DNA obtained from ZeptoMetrix Corporation (NY, United States) was used as the positive control for establishing the analytical sensitivity of SIBA, LAMP, and PCR singleplexed NG assays. Analytical specificity of SIBA, LAMP, and PCR singleplexed assays was established by challenging the reactions with a mixture of DNA from various microorganisms (10^3^ copies from each microorganism per reaction). The specificity of SIBA, LAMP, and PCR NG assays were further challenged with 10^5^ copies of DNA from *Neisseria meningitidis*, *Neisseria flavescens*, *Neisseria subflava*, and *Neisseria sicca*. CT LGV-II Heat-Inactivated Elementary Bodies (EB) obtained from Advanced Biotechnologies (MD, United States) and NG strain FA1090 (ATCC 700825) were used to establish the performance of multiplexed CT/NG/IC SIBA and PCR assays. EB and NG cell lysates were prepared by heating at 90 °C for 10 minutes. An appropriate amount of lysate was then added directly to the multiplexed CT/NG/IC reactions. The microorganisms used in this study are listed in Table 3. AMPLIRUN® TOTAL CT/NG CONTROL (Vircell, Spain) samples were used to establish the performance of mSIBA for the detection of CT and NG cells in the presence of clinical sample matrix. One ml of the clinical sample was centrifuged at 10,000 rpm for 10 minutes. The supernatant was then discarded and the pellet was re-suspended in 100 μl of water and 2 μl was added to the mSIBA reaction.

### SIBA assays

SIBA assays for CT and NG were designed to amplify specific sequences from the CT cryptic plasmid and *porA* gene, respectively. The SIBA assays consisted of two amplification primers and an invasion oligonucleotide (IO)^6^. An IC assay was also designed to amplify an artificial DNA sequence that shared homology with the same target sequence as the CT assay. The IC used the same forward primer and IO as the CT assay, but the reverse primer differed.

SIBA reactions were performed using a commercial SIBA reagent kit with UvsX and gp32 enzymes at 0.25 mg/ml each (Orion Diagnostica Oy, Finland). NG assay reactions were performed with the addition of 2 U of the restriction endonuclease *Mly*I (New England Biolabs,United States). For multiplexed CT/NG/IC reactions, an additional 75 mM sucrose and 0.25 U sucrose phosphorylase were included into the SIBA reaction. All reactions contained 14 mM magnesium acetate, except for the NG singleplexed reactions, in which 10 mM magnesium acetate was used.

SIBA amplification was detected using either SYBR Green 1 (1:100,000 dilution) or target-specific probes. The probes were short dual-labeled oligonucleotides (12–14 nt) containing a mixture of DNA/LNA bases and homologous to the 5′ region of the reverse primers. SIBA reactions were incubated at 40 °C for 60 minutes, and fluorescence readings were taken at 60-second intervals on an Agilent MX Pro 3005P (Agilent Technologies, Inc., CA, United States). After incubation for 60 minutes, the instrument was set to run a melt curve from 40 °C to 95 °C in order to further assess the specificity of SIBA reactions. For all reactions, forward and reverse primers and the IO were used at 200 nM final concentrations, except for the IC assay, in which the reverse primer was used at 100 nM final concentration. Probes for CT, NG, and IC assays were used at 150, 50 and 100 nM, respectively.

### LAMP assays

The LAMP assay for NG was designed to amplify specific sequence from the *porA* gene using PrimerExplorer Version 4 ( http://primerexplorer.jp/e/). Final primer concentrations for F3 and B3 were 0.2 μM, whereas BiP and FiP were used at 1.6 μM. LAMP reactions were also set up for previously published assays for the CT cryptic plasmid^19^ and NG *ORF1* of the glutamine synthetase *(glnA)* gene^7^. Final primer concentrations for the previously published CT cryptic plasmid and NG LAMP assays were as specified by the authors. All LAMP primers are listed in Table 4. LAMP reactions were performed using a commercial LAMP reagent kit with OptiGene Isothermal Mastermix ISO-100 (OptiGene, UK). The reactions were conducted at 64 °C for 60 minutes according to the manufacturer’s instructions. Real-time detection of LAMP reactions was monitored on an Agilent MX Pro 3005P. Fluorescence readings were taken once every minute.

### PCR assays

The results of previously published real-time PCR assays validated for detection of CT cryptic plasmid and NG *porA* in urine were compared with those of the SIBA assays^8^. The reactions were performed using iTaq Universal Probes Supermix (Bio-Rad, UK). For singleplexed CT and NG reactions, the primers and probes were used at 400 nM and 200 nM final concentrations, respectively. Singleplexed reactions were run with a 3 minute denaturation step at 95 °C, followed by 50 cycles of 95 °C for 15 seconds and 60 °C for 60 seconds. For multiplexed CT/NG reactions, primers and probe were used at 250 nM and 100 nM final concentrations, respectively, and IC primers were used at 100 nM final concentration. Multiplexed reactions were run with a 5 minute denaturation step at 95 °C, followed by 50 cycles of 95 °C for 15 seconds, 60 °C for 60 seconds, and 72 °C for 1 second and a final incubation at 40 °C for 30 seconds.

## Additional Information

**How to cite this article**: Eboigbodin, K. E. and Hoser, M. J. Multiplex Strand Invasion Based Amplification (mSIBA) assayfor detection of *Chlamydia trachomatis* and *Neisseria gonorrhoeae*. *Sci. Rep.* **6**, 20487; doi: 10.1038/srep20487 (2016).

## Supplementary Material

Supplementary Information

## Figures and Tables

**Table 1:** Sensitivities of SIBA, LAMP, and PCR assays for detection of *Neisseria gonorrhoeae*.

	SIBA* *porA*	LAMP* *porA*	LAMP* ORF1	PCR¶ *porA*
**Sensitivity** *N. gonorrhoeae* genome copy number				
2 × 10^5^	+	+	+	+
2 × 10^4^	+	+	+	+
2 × 10^3^	+	+	+	+
2 × 10^2^	+	+	+	+
2 × 10^1^	+	+	+	+
**Specificity**				
*Neisseria* strains (Non-gonococcal)	-	+	+	-
Non-target bacterial mix	-	-	-	-

^*^SIBA and LAMP reactions were detected using intercalating dyes. ^¶^PCR reactions were detected with Taqman probes.

**Table 2:** Sensitivity of SIBA, LAMP and PCR assay for the detection of *Chlamydia trachomatis*.

	SIBA* Cryptic plasmid	LAMP* Cryptic plasmid	PCR¶ Cryptic plasmid
**Sensitivity** Plasmid copy number			
2 × 10^5^	+	+	+
2 × 10^4^	+	+	+
2 × 10^3^	+	+	+
2 × 10^2^	+	+	+
2 × 10^1^	+	+	+
**Specificity**			
*Neisseria* strains (Non-gonococcal)	-	-	-
Non-target bacterial mix	-	-	-

^*^SIBA and LAMP reactions were detected using intercalating dyes. ^¶^PCR reactions were detected with Taqman probes.

**Table 3:** List of microorganisms used for determination of analytical specificity.

Non-gonococcal *Neisseria* strains	*Neisseria meningitidis*
	*Neisseria flavescens*
	*Neisseria subflava*
	*Neisseria sicca*
Non-*Neisseria* strains	*Pseudomonas aeruginosa*
	*Candida albicans*
	*Aeromonas hydrophila*
	*Escherichia coli*
	*Klebsiella pneumoniae* subsp*. pneumoniae*
	*Enterobacter cloacae*
	*Serratia marcescens*
	*Proteus mirabilis*
	*Staphylococcus aureus*
	*Enterococcus faecium* VanA

**Table 4:** Oligonucleotides used in this study.

Method	Target	Primer/probes/IO	Sequence	Reference
**SIBA**				
	CT-plasmid	F-primer R-primer IO Probe-ROX	TTATCGACTGGGTGATTA CTTTCTGGCCAAGAATTAT TCCTCCTCTTCCTTGATTACAGCAGCTGCGAAAAAGAGACGAAAAmU mUmAmAmCmUmAmAmGmGmAmUmAmAmUmU(InvdT) (ROX)CC+T+TT+C+TGG+C+C+AA+G(IABkFQ)	In this study
	NG-*porA*	F-primer R-primer IO Probe-CY5	GCAAGTCCGCCTATACGCCTG CCGACAACAGCCGGAACT TTCTCCTTACACTCGCCTGCTACTTTCACGCTGGAAAGTAATCAG mAmUmGmAmAmAmCmCmAmGmUmUmCmCmGmA(InvdT) (CY5)+C+C+G+A+C+A+AC+AG+C+C(IAbRQSp)	
	IC	R-primer Probe-FAM Template	TCTCGGTCAATATATTTA (FAM)+T+C+G+G+T+C+A+A+T+A+T(DABCYL) ATTTATCGACTGGGTGATTACAGCAGCTGCGAAAAAGAGACGAAAAT TAACTAATAAATATATTGACCGAGA	
**LAMP**				
	CT-plasmid	LoopB LoopF LampB LampF B3 F3	CGAGCAGCAAGCTATATT AAACTCTTGCAGATTCATA GACCGAAGGTACTAAACAAGTTTTTTTGTTTAGGAATCTTGTTAAGGGCGCATCTAGGATTAGATTAGATTTTATTGGTCTATTGTCCTTGG TATTCCTTGAGTCATCC GATCATATCGAGGATCTT	19
	NG-*ORF1*	LampB LampF B3 F3	TGTAGTAGAGCGCGGTATCGGACGGTCAAAACCTGTTCGCA CAAACACGCCAAAGCCCTGAACGCACGAGGCGTTTGTAGG GCACGTCCACCAATCCATT TGGTCGGTGCTTCAAAGTG	7
	NG-*porA*	LampB LampF B3 F3	TCCCCCGGATTTTCCGGTTTCAGTAGCAGGCGTATAGGCG GTAACGCACGGAAACCGGCAGTGGCTTCGCAATTGGGTA CCGGAACTGGTTTCATCTGA TTGATCCTTGGGACAGCAAT	In this study
**PCR**				
	CT-plasmid	F-primer R-primer Probe-FAM	AACCAAGGTCGATGTGATAG TCAGATAATTGGCGATTCTT (FAM)CGAACTCATCGGCGATAAGG(BHQ1)	8,11
	NG-*porA*	F-primer R-primer Probe-ROX	CAGCATTCAATTTGTTCCGAGTC GAACTGGTTTCATCTGATTACTTTCCA (ROX)CGCCTATACGCCTGCTACTTTCACGC(BHQ2)	
	IC control	F-primer R-primer Probe-CY5 Template	GTGCTCACACCAGTTGCCGC GCTTGGCAGCTCGCATCTCG (CY5)ATTGTGTGGGTGTGGTGTGGGTGTGTGC(BHQ3) GTGCTCACACCAGTTGCCGCGGAAAGTATGTGGAATGTTAACACACCCACACCACACCCACACACGTGTTGGATCAATTTCGAGATGCGAGCTGCCAAGC	

m = 2’-O-methyl RNA bases; InvdT = inverted dT, + = lock nucleic acid bases; IABkFQ = Iowa black FQ quencher; IAbRQSp = Iowa black RQ quencher; BHQ1 = black hole quencher 1; BHQ2 = black hole quencher 2; BHQ3 = black hole quencher 3.
