# Supplementary material for: Multiplex Strand Invasion Based Amplification (mSIBA) assay for detection of Chlamydia trachomatis and Neisseria gonorrhoeae
Source: Sci Rep. 2016 Feb 3;6:20487. doi: 10.1038/srep20487 (PMC4738295; doi:10.1038/srep20487)
Supplement: Supplementary Information [file srep20487-s1.pdf]

Multiplex Strand Invasion Based Amplification (mSIBA) assay

for detection of *Chlamydia trachomatis* and *Neisseria gonorrhoeae*

Kevin E Eboigbodin[1] and Mark J Hoser[2],

[1]Research and Development, Orion Diagnostica, Espoo, Finland

[2]Molecular Biology, GeneForm Technologies, Broadstairs, United Kingdom

To whom correspondence should be addressed: Mark J. Hoser, Molecular Biology, GeneForm Technologies, Broadstairs, United Kingdom,

Tel:+44797096004, E-mail: markhoser @gmail.com

Supporting information S1

A. SIBA porA assay

```
                                                                                                    F-primer        IO
N. gonorrhoeae  GATGCCAGCAAAGCCATTGATCCTTGGGACAGCAATAATAATGTGGCTTCGCAATTGGGTATTTTCAAACGCCAC---GACGGTATGCCGGTTTCCGTGC 97
N. meningitidis .........C.............................G...................................---....AC..............A. 97

N. gonorrhoeae  GTTACGATTCCCCCGGATTTTCCGGTTTCAGCGGCAGCATTCAATTTGTTCCGAGTCAAAACAGCAAGTCCGCCTATACGCCTGCTACTTTCACGCTGGA 197
N. meningitidis .C.............A......................G.......C.......TC..........................G...TA..AT..TAA.A. 197

                      IO
                                        R-primer
N. gonorrhoeae  ---AAGTAATCAGATGAAACCAGTTCCGGCTGTTGTCGGCAAGCCGGGGTCGGATGTGTATTATGCCGGTCTGAATTACAAAAATGGCGGCTTTTTCGGA 294
N. meningitidis TAC..AC...A.TC.T.CT.TC.......................C..A.........................................T...GC...G 297
```

A. PCR porA assay

```
N. gonorrhoeae  GATGCCAGCAAAGCCATTGATCCTTGGGACAGCAATAATAATGTGGCTTCGCAATTGGGTATTTTCAAACGCCAC---GACGGTATGCCGGTTTCCGTGC 97
N. meningitidis .........C.............................G...................................---....AC..............A. 97

                                                     F-primer                          Probe
N. gonorrhoeae  GTTACGATTCCCCCGGATTTTCCGGTTTCAGCGGCAGCATTCAATTTGTTCCGAGTCAAAACAGCAAGTCCGCCTATACGCCTGCTACTTTCACGCTGGA 197
N. meningitidis .C.............A......................G.......C.......TC..........................G...TA..AT..TAA.A. 197

                         R-primer
N. gonorrhoeae  ---AAGTAATCAGATGAAACCAGTTCCGGCTGTTGTCGGCAAGCCGGGGTCGGATGTGTATTATGCCGGTCTGAATTACAAAAATGGCGGCTTTTTCGGA 294
N. meningitidis TAC..AC...A.TC.T.CT.TC.......................C..A.........................................T...GC...G 297
```

B. LAMP porA assay

```
                                              F3                          F2                                   F1c
N. gonorrhoeae   GATGCCAGCAAAGCCATTGATCCTTGGGACAGCAATAATAATGTGGCTTCGCAATTGGGTATTTTCAAACGCCAC---GACGGTATGCCGGTTTCCGTGC 97
N. meningitidis  .........C.............................G...................................---....AC..............A. 97

                                    B1c                                                          B2
N. gonorrhoeae   GTTACGATTCCCCCGGATTTTCCGGTTTCAGCGGCAGCATTCAATTGTTCCGAGTCAAAACAGCAAGTCCGCCTATACGCCTGCTACTTTCACGCTGGA 197
N. meningitidis  .C.............A......................G.......C.......TC..........................G...TA..AT..TAA.A. 197

                                     B3
N. gonorrhoeae   ---AAGTAATCAGATGAAACCAGTTCCGGCTGTTGTCGGCAAGCCGGGGTCGGATGTGTATTATGCCGGTCTGAATTACAAAAATGGCGGCTTTTTCGGA 294
N. meningitidis  TAC..AC...A.TC.T.CT.TC.......................C..A.........................................T...GC...G 297
```

FIP contain F1c and F2 sequence
BIP contain B1c and B2 sequence

Figure S1 Alignment map showing the location of SIBA, LAMP and PCR NG oligonucleotides in relation to porA pseudogene from *Neisseria gonorrhoeae* (NG) and *Neisseria meningitides* (NM). NG strain FA1090, accession number AJ223447.1 (position 597-890) and NM strain MC58 accession number AF226344.1 (position 406-702)

Supporting information S2

Affinity of Single-Stranded binding protein to LNA bases

The effect of single-stranded binding protein (gp32) on the signal produced by *Chlamydia trachomatis* (CT) and *Neisseria gonorrhoeae* (NG) dual labeled probes was investigated. The probes were composed of natural DNA or a mixture of LNA and DNA bases. 100 nM of probes were incubated in the presence or absence of 400 mM complementary template and 250 ng/ml of gp32 (concentration used in SIBA reactions) in a buffer containing 20 mM Tris-acetate pH 7.9, 50 mM potassium acetate, 10 mM magnesium acetate and 1 mM DTT. The fluorescence signals of these probes were measured after 10 minutes incubation at 40 degrees centigrade using the Agilent MX Pro 3005P (Agilent Technologies, Inc., CA, United States). Incubation of the dual labeled probes with their complementary template led to an increase in fluorescence signal compared with signal produced in the absence of the complementary template. In the absence of a complementary template, the presence of single-stranded binding protein, gp32 lead to dramatic increase in background fluorescence signal for dual labeled probes containing only DNA bases (CT-probe-LNA0 and GC-probe-LNA0). Consequently, a low signal-to-background florescence was observed upon hybridisation of dual labeled probes (only containing DNA bases) with the complementary template in the presence of gp32 (Figure S2). The high background signal induced by the presence of gp32, could elicit a false fluorescence signal especially when used for the detection of low amount of complementary template. Dual labeled probes containing only DNA bases are undesirable for real-time detection of a complementary template in reactions where a single-stranded binding protein is present (e.g. SIBA reaction). In contrast, dual labeled probes having identical sequence but composed of three or more LNA bases displayed lower background fluorescence in presence of gp32 alone (without the complementary template, Table S1). The background fluorescence decreases as the number of LNA bases within the dual labeled probes increase. These chimeric DNA-LNA dual labeled probes displayed increase in fluorescence

signal in the presence of gp32 and their complementary template. Consequently, a higher signal-to-background fluorescence was observed upon hybridisation of dual labeled probes containing LNA bases with the complementary template in the presence of gp32. CT-probe-LNA8 (8 LNA bases) and GC-probe-LNA10 (10 LNA bases) were the probes used for the detection of CT and NG in SIBA reactions respectively.

Table S1 Sequences, signal-to background ratio, *Chlamydia trachomatis* (CT) and *Neisseria gonorrhoeae* (NG) probes in the presence of single strand DNA binding protein and complementary template.

**Probes**	**Sequence**	**no.of LNA bases**	**Signal-to-background ratio**
GC-probe-LNA0	/5CY5/CCGACAACAGCC/3IAbRQSp/	0	1.3
GC-probe-LNA3	/5CY5/CCG+AC+AAC+AGCC/3IAbRQSp/	3	2.5
GC-probe-LNA7	/5CY5/+CC+GA+C+A+AC+AGC+C/3IAbRQSp/	7	4.7
GC-probe-LNA10	/5CY5/+C+C+G+A+C+A+AC+AG+C+C/3IAbRQSp/	10	5.4
CT-probe –LNA0	/56-ROXN/CCTTTCTGGCCAAG /3IABkFQ/	0	2.2
CT-probe-LNA2	/56-ROXN/CCT+TTCTGG+CCAAG /3IABkFQ	2	2.8
CT-probe-LNA8	/56-ROXN/CC+T+TT+C+TGG+C+C+AA+G /3IABkFQ/	8	5.6

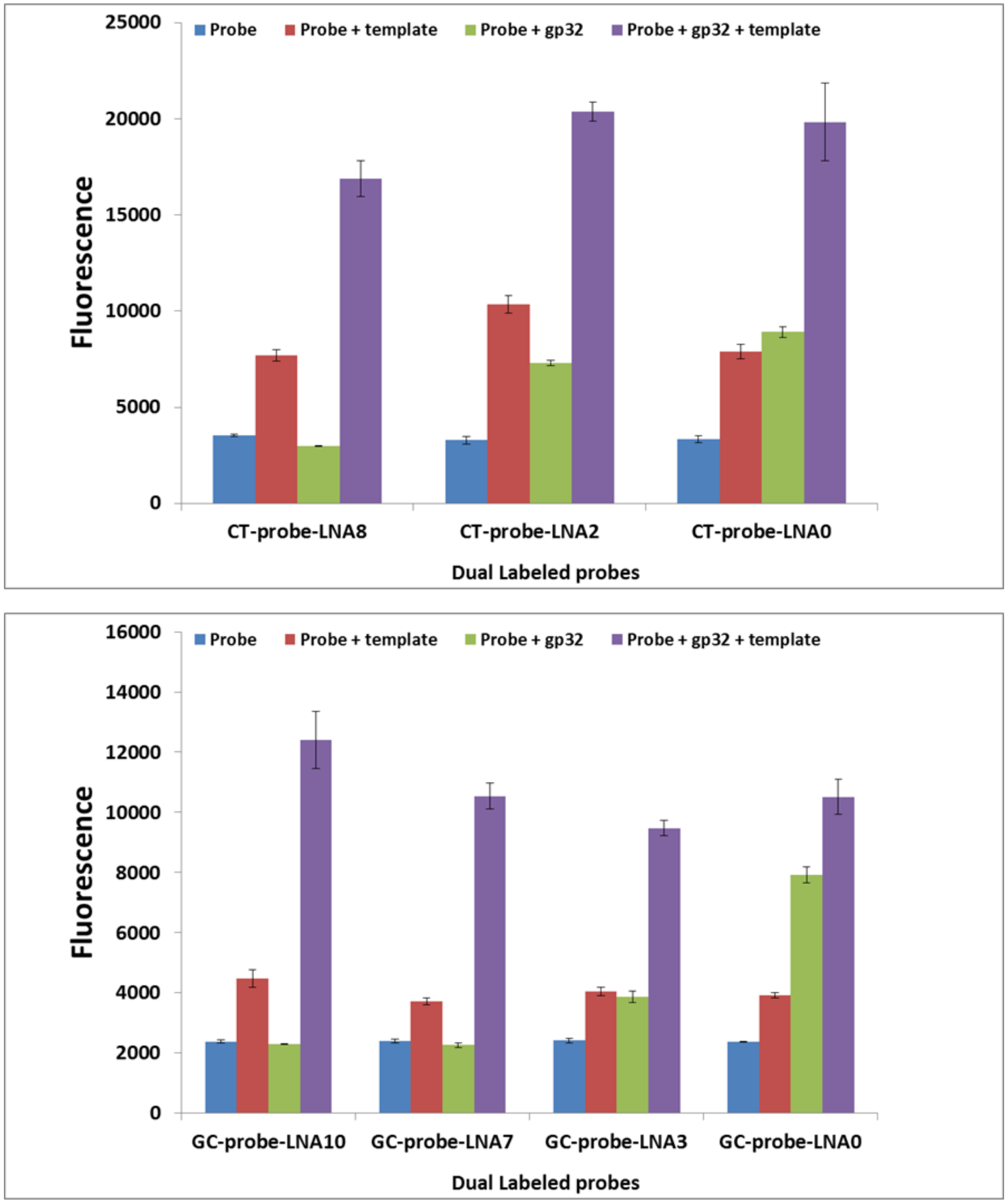

Figure S2 Effect of single-stranded DNA binding protein (T4 gp32) on signal from CT and NG dual labeled probes. Probes either contained natural DNA bases or DNA/LNA chimeric bases. + Equal LNA bases. CT-probe-LNA8 and GC-probe-LNA10 were the probes used for the detection of CT and NG SIBA reactions. Fluorescence readings are based on average of 6 replicates

Supporting information S3

Detection of *Chlamydia trachomatis* (CT) and *Neisseria gonorrhoeae* (NG) from clinical control sample

The performance of mSIBA for the detection of *Chlamydia trachomatis* (CT) and *Neisseria gonorrhoeae* (NG) was further evaluated using a commercially available clinical control samples containing low number of CT and NG cells (AMPLIRUN® TOTAL CT/NG CONTROL URINE, Vircell, Spain). Samples (1 ml) were centrifuged at 10,000 rpm for 10 minutes and the supernatant was discarded. The pellet was re-suspended in 100 µl of water and 2 µl of the suspension was then added to the mSIBA reaction. The samples were reproducibly detected by mSIBA. The amplification curves can be seen in figure Ssupporting information S3

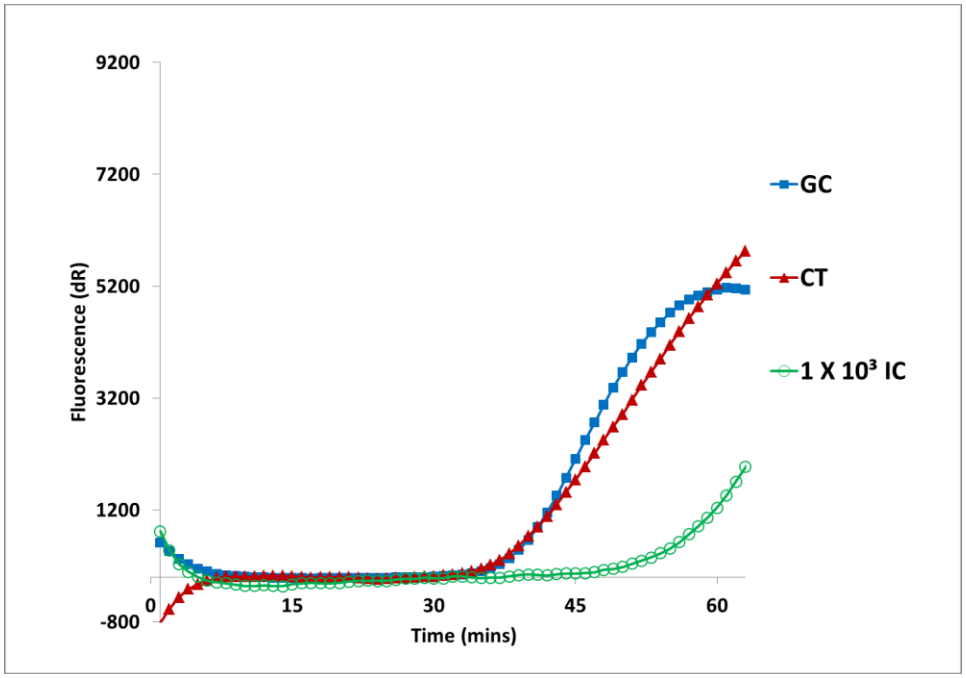

Figure S3 Detection of *Chlamydia trachomatis* (CT) and *Neisseria gonorrhoeae* (NG) from clinical control sample
